# Mental Imagery Skills in Alcohol-Dependent Subjects and Their Associations With Cognitive Performance: An Exploratory Study During Residential Rehabilitation

**DOI:** 10.3389/fpsyt.2021.741900

**Published:** 2021-11-29

**Authors:** Marcella Ottonello, Elisa Torselli, Stefano Caneva, Elena Fiabane, Claudio Vassallo, Caterina Pistarini

**Affiliations:** ^1^Department of Physical and Rehabilitation Medicine of Genova Nervi Institute, Istituti Clinici Scientifici (ICS) Maugeri Spa SB, Genoa, Italy; ^2^School of Psychotherapy, Miller Institute for Behavioral and Cognitive Therapy, Genoa, Italy; ^3^Department of Neuroscience, Rehabilitation, Ophthalmology, Genetics, Maternal and Child Health, University of Genoa, Genoa, Italy; ^4^Department of Neurorehabilitation Medicine of Pavia Institute, Istituti Clinici Scientifici (ICS) Maugeri, Istituti di Ricovero e Cura a Carattere Scientifico (IRCCS) Spa SB, Pavia, Italy

**Keywords:** mental imagery (MI), alcohol-dependence, cognitive rehabilitation, mental disorders, alcohol detoxification

## Abstract

People in alcohol-detoxification experience deficits in motor and non-motor functions including cognitive performance. Imagery, the cognitive process of generating visual, auditory or kinesthetic experiences in the mind without the presence of external stimuli, has been little studied in Alcohol Use Disorders (AUD). This pilot study aims to observe the cognitive abilities useful for the inspection, maintenance, generation and manipulation of images in these patients during residential rehabilitation and investigate the relationships with their cognitive performance. Thirty-six subjects who completed the 28-day rehabilitation program for alcohol addiction, completed the Mental Imagery Test (MIT) and Neuropsychological Battery (ENB-2). The global score at MIT did not show pathological scores. The 11.1% of AUD patients showed an impaired global score in the cognitive performance and the 5.7% with scoring at limits of norm. Significant correlations were found between Mental Imagery abilities and ENB-2 subscale and stepwise regression analysis showed the close association between the ability of imagery (Mental Imagery Test) and the overall cognitive performance (ENB-2) in alcohol dependent patients and this relationship is stronger than other cognitive tasks.

## Introduction

Alcohol abuse is associated with significant alteration of brain structure, physiology and functions. Neuroimaging studies provided evidence for ethanol-induced multiple brain lesions, that can account for cognitive and motor impairments (1, 2). The cognitive deficits mainly affect executive functions, episodic memory and visuospatial abilities. Specifically, individuals with alcohol use disorders (AUD) are at risk for executive cognitive functioning impairments as well as damage to the frontal brain (3). Although the maintenance of sobriety is associated with cognitive recovery, some deficits may persist and interfere with the motivation process of patients to change their addictive behavior (4). The literature showed that after alcohol detoxification, 50–80% of the recently abstinent subjects present neuropsychological impairments (5–7). Alcohol-related brain damage is characterized by a brain volume deficit, a dilatation of the ventricles and an increased cerebrospinal fluid quantity, involving several brain regions: cerebellum, corpus callosum, hippocampus, thalamus, amygdala, and frontal cortices (8). These neuroanatomical alterations can account for neuropsychological impairments involving attention, executive functions, memory, visuospatial and motor skills. Cognitive and neuropsychological deficits may interfere with the ability to learn, retain or use new information, affecting on treatment outcome in AUD, through an effect on change processes and mechanism of change (9). Alterations of episodic memory in AUD subjects is related to limited learning abilities, impairments of encoding and recollection deficits, while storage capacities appeared to be preserved (4). The executive functions also play a key role on memory impairment. These functions are known to contribute to the strategic organization of information in order to facilitate encoding and recovery in memory, integrating diverse types of information (factual, temporal, spatial) into a meaningful representation. Disorders of inhibition, flexibility, categorization, organization and planning have been frequently found in AUD subjects with alterations of executive functions (10). Finally, visuospatial cognition is also compromised in detoxified alcoholics, including functions such as scanning and using visual images and more complex operations of manipulation, storing and retrieving of visuospatial information (11). Although many studies show that the recovery of different cognitive functions depends on the type of function and some cognitive deficits are reversible after few weeks after abstinence, for others, residual deficits persist over time (12). The literature suggested that the cognitive impairment may reduce the effectiveness of psychological treatments in AUD and that cognitive dysfunctions are directly related to compliance with treatment and maintenance of abstinence (13).

Cognitive Behavioral Therapy (CBT) is one of the most extensively evaluated interventions for treating alcohol dependence and it was the psychotherapeutic intervention carried out during the residential rehabilitation in this current study. This treatment requires the planning and the learning of new behavioral procedures and provides skills training to explore functional alternatives to the use of alcohol (14). These procedural learning abilities are impaired in AUD and may be the consequence of a delayed transition from the cognitive phase of procedural learning to the later stages (associative and autonomous). Indeed, according to the Adaptive Control of Thoughts model (15), the encoding of a new cognitive procedure occurs in three successive phases: the cognitive phase, which is a highly controlled stage, the individual performances are linked to general intelligence, and episodic and working memory, as well as to executive functions. The autonomous phase is characterized by psychomotor abilities and procedural memory *per se*. The associative phase is the transition phase between the cognitive and autonomous phases. The activation of occipital regions during these later phases suggested the intervention of mental imagery (16). Mental imagery is an important part of information processing performed during interpreting and involved in reasoning and problem solving (17).

The term “Mental Imagery” (MI) refers to representations that give rise to the experience of viewing a stimulus without a direct external stimulus being present in the sensorial systems.

Recent behavioral, brain imaging, and clinical research has reshaped our understanding of MI as a depictive internal representation that functions like a weak form of perception. Brain imaging research demonstrated that neural representations of mental and perceptual images resemble each other right from the primary visual cortex (V1) (18). Results from these new studies highlight the role of MI in perception, cognition, and mental health (19, 20).

MI is multi-sensory and can be classified into any sensory mode. Visual, spatial and motor imagery are the most discussed mental approaches in the literature (21). Many studies have shown that the training in MI leads to substantial improvements in memory performance in adults. Usually, pictures are better remembered than words: image codes activate primary visual cortex, while verbal codes usually activate prefrontal and temporoparietal areas (22). Imagery plays an important role in memory, abstract reasoning, skill learning and spatial reasoning (23). The ability to work with images is different in people's use and these individual differences are accentuated by the consequences of brain damage (including working memory) (24). MI has also been applied to improve motor function after stroke and results showed positive effects on relearning of lost functions and performance (25). Alcohol abuse, however, is associated with extensive dysfunctions of the brain and specifically of the prefrontal cortex, involving executive processes such attention, inhibitory control, working memory, and cognitive flexibility. Several studies underlie the key role of MI in various psychological disorders and that Imagery can be implicated in the onset and chronicity of affective disorders (26). Finally, a growing body of scientific literature highlights its effectiveness in enhancing motor and cognitive aspects of performance in a variety of subjects: athletes and people with neurodegenerative disorders, such as Parkinson's and Alzheimer's disease (22, 27–29). To the best of our knowledge, very few studies (30) explored MI among AUD subjects. The rationale of the research was to evaluate the performance in the skills of MI in this study population and its relationships with the other cognitive abilities.

Therefore, the primary aim of the current study was to describe the ability in MI among AUD subjects in the early stages of a 28-day residential rehabilitation program. The secondary aim was to investigate the relation of the MI with the cognitive functioning in this population, considering the effects of alcohol abuse on the brain and cognitive performance. In fact, cognitive assessment tests involve visualization components that interact with verbal and memory functions and therefore can affect overall cognitive performance. This pilot study aims to examine if and how much the specific skills of visualization and image processing are involved in the global cognitive functioning in AUD subjects.

## Materials and Methods

### Participants

Thirty-six subjects who completed the 28-days rehabilitation program for alcohol addiction by Department of Physical & Rehabilitation Medicine of ICS Maugeri Spa SB (Institute of Genoa) were included in this pilot study. The subjects admitted to residential rehabilitation held desire to stop drinking and a willingness to undergo an intensive therapeutic program characterized by a short duration and a high intensity of care (31, 32). Inclusion criteria to the study were as follows: age >18 years, current diagnosis of alcohol dependence according to the *Diagnostic and Statistical Manual of Mental Disorders*, Fifth Edition, (DSM-5) (33), absence of dementia (evaluated with Mini–Mental State Examination ≥24), understanding of the Italian language (if foreigners subjects) and participation in ≥80% of the proposed rehabilitation sessions. The exclusion criteria were as follows: a history of other brain damage (stroke, neoplasm or neurological disease), absence of severe psychiatric conditions (e.g., psychosis or suicidal ideations in the last month). The rehabilitation program was standardized: the physical rehabilitation included 1-h/day group gymnastic with aerobic and anaerobic exercises and 1-h/day group relaxation techniques. Psychological intervention included:

1-h group therapy sessions twice daily regarding the maintenance of abstinence, the education of alcohol-related risks, motivation to change, CBT training (coping skill training) and relapse prevention.

Of the eligible 40 participants, 2 (5%) were excluded from the study for dropout of treatment and did not complete the rehabilitation program and 2 (5%) refused to participate in the research.

A-priori power analysis (G^*^Power) (34) was performed based on a correlational model with the following parameters: two-tailed α = 0.05; a medium-to-large expected effect size of 0.45; 1-β = 0.80. This analysis yielded a minimum sample size required of *N* = 33.

Following admission to the rehabilitation unit, the participants were assessed by an addiction-medicine physician through a short interview to identify the severity of alcohol dependence using the Alcohol Use Disorders Identification Test (AUDIT); moreover, personal information, such as age, level of education, work status, period of alcohol dependence, possible presence of other drug dependence, or psychiatric comorbidities were collected. The neuropsychological battery and MI Test were administrated at the beginning of residential treatment after 7 days of complete detoxification. Two trained neuropsychologists, not aware of the aims of the study, carried out MIT test and neuropsychological assessment, according to standardized procedures, The residential treatment included: evaluation and treatment of acute withdrawal symptoms, cognitive behavioral therapy, group activities, physiotherapy, and education on alcohol related risk and skills training (31, 35).

The research was conducted in accordance with the Declaration of Helsinki. Regional Ethics approval was obtained from the Institute before starting the study (CER Liguria 387/2018) and a written informed consent was obtained from all participants.

All data collected in this study derived from tools and treatments used in clinical practice.

### Measures

#### Neuropsychological Assessment

Brief Neuropsychological Examination 2 (ENB-2): It is a comprehensive neuropsychological battery (36) standardized for the Italian population. The battery investigates with 16 subtests the cognitive areas of attention: Trail Making Test—A (TMT- A), executive functioning (word phonemic fluency, clock drawing, abstract reasoning, TMT- B), praxis abilities (ideative and ideomotor praxis tests), language (Token test), memory (digit span, immediate and delayed recall in prose memory, interference memory at 10 and 30 sec) and visuo-spatial abilities (overlapping figures, spontaneous drawing, copy drawing).

The scoring system yields a score for each subtest and a total score (the Global Cognitive Index (GCI), describing the global cognitive profile. The scores are classified in three categories: below average (impaired), at the limit, and average (normative), using the 5th percentile as cut-off scores. The battery showed to have good psychometric properties, including adequate test-retest reliability and differential validity in discriminating normative and clinical groups (36–38).

### Imagery Assessment

#### Mental Imagery Test (MIT)

It consists of a series of subtests that include different components of MI skills: maintenance, inspection, generation, and manipulation (39). These are the eight tasks:

- Visualizing letters (generation): the individuals are invited, to imagine some upper-case letters without seeing the stimuli and say which have curled parts (e.g., A, P, or R; not L, M, or N).
- Brooks “F” test (inspection): It requires to examine mentally the contour of a letter F recalling in sequence the direction (internal/external) of its corners.
- Clock (generation/manipulation): the task requires to visualize a clock with hands indicating 10 min past 10:00, then saying what time the clock, reflected in a mirror, will show after 10 min.
- Cube (maintenance): the image of a large cube, made up of nine small cubes per face (with external faces colored), is shown for 30 s; After the stimulus is removed, the subject is asked to state how many small cubes have three, two, one, or none external (colored faces).
- Subtraction of parts (manipulation): a digital display with the number 88 collected of small segments is shown for 10 s. Then, is asked to deduce in imagination parts from figures.
- Mental exploration of a map (inspection): it is asked to evaluate the distance between elements present in a map of an island: a house, a church, a lake, and a wood located on it.
-Imagined paths (manipulation): subject is asked to imagine a small ball moving in dissimilar directions, according to the instructions of examiner and saying if at the end of the route the ball will end up above or below the initial point, or at the same level.
-Mental representation of shapes of objects (mantenance): the individual visualizes the shape of 20 concrete objects and then selects if the object has a taller or larger form.

A total score of MI can be found by the sum of the scores in the single subtests. It has a good reliability (Cronbach's alpha for this score of imagery ranging from 0.75 to 0.78 according to age). A confirmatory factor analysis showed the monodimensionality of the measure containing the eight tasks. The selection of the tasks was designed at expressing all the functions implicated in imagery, agreeing to the classification proposed by Pearson et al. (26) and the test is able to discriminate the imaginative performances both in normal subjects and with mental deterioration (39).

#### Comparison Tasks

MIT Battery also includes comparison cognitive tasks with the aim of discriminating MI abilities from cognitive skills that do not involve active generation and /or transformation of mental images. Comparative tasks allow better discriminate deficits than other cognitive performances (ENB-2) in basic cognitive functions since they assess cognitive functions (attention, memory and perception) without an active image processing in the working memory. The comparison tasks are the following:

Forward and backward digit-span (analogous as WISC digit-span subtest). The individual hears progressively increasing digit series and has to repeat them in direct or backward order. Attentional tasks in which working memory is involved.

Memory of objects and of position of objects. The subject, after seeing seven concrete objects for 30,” is invited to remember as many of them as possible in a free recall. In the next task, the subject has to recall the position in a matrix of six objects previously seen for 30.”

Visual-spatial memory test. Subjects are presented for 10” with a matrix in which a number of cells are filled, and after are required to remember where they were located using a blank matrix. Six matrices are presented. These tasks of memory of position, require a passive maintenance of spatial position in working memory, without transformations of mental images.

Mirror reproduction of spatial position. The patient is requested to copy a model in a mirror position, though actually seeing the model. This test of copy involves spatial perception without active alteration of images.

### Alcohol Use Disorders Identification Test (AUDIT)

The Alcohol Use Disorders Identification Test (AUDIT) is a 10-item questionnaire developed by the World Health Organization (WHO) to assess alcohol consumption, drinking behaviors and alcohol- related problems (40). Participants are asked to choose one of five statements (per item) that most applies to their alcoholic habits over the past year. Each item receives a score of 0–4, with a total possible score of 40 (hazardous or harmful alcohol use). A score of 8 or greater is generally accepted as indicating an alcohol problem. It has become a widely used instrument and it possesses good validity and reliability (41).

### Statistical Analysis

A descriptive analysis was used to present the basic characteristics (socio-demographic and clinical) of the study participants. Skewness, Kurtosis and Shapiro-Wilk tests are used to determine if MIT and ENB-2 are normally distributed. Only four subscales of the ENB-2 did not meet the normality criteria and in this case, we used non-parametric statistics. Pearson's or Spearman's correlation analysis was performed to examine bivariate correlations among neuropsychological and MI scales.

A *p*-value of 0.05 was considered statistically significant. All statistical analyses were conducted using STATA 14.0 software. The AUDIT scale was dichotomized considering the median of the scores' distribution, in high level (≤ 33) or very high level (≥34) of severity of alcohol dependence (42). Multiple linear regression was used to test the importance of MI on global cognitive performance measured by ENB-2.

Participants were stratified into groups by sex, level of education, work status (employed, unemployed), substance dependence (pure abusers of alcohol vs. polyabusers), type of intervention (drugs plus psychotherapy sessions vs. psychotherapy sessions only) and previous recovery (presence or absence of previous recovery for alcohol dependence).

## Results

### Descriptive Analysis

Participants had a mean age of 53.03 ± 9.39 years; the majority of the sample was males (58.3%), employed (72.2%) with a middle-high education level (77.8%) and a history of more of 10 years of alcohol dependence (66.7%) without polysubstance dependence (77.8%). The socio-demographic and clinical characteristics of the sample are shown in Table 1.

**Table 1:** Sociodemographic and clinical characteristics of the sample (*N* = 36).

**Variables**		**N (%)**
Age (years)	30–50	15 (41.7)
	≥51	21 (58.3)
Gender	Male	21 (58.3)
	Female	15 (41.7)
Education (years)	≤ 8	8 (22.2)
	≥9	28 (77.8)
Work Status	Employed	26 (72.2)
	Unemployed	10 (27.8)
AUDIT	≤ 33	24 (66.7)
	≥ 34	12 (33.3)
Alcohol dependence (years)	2–9	12 (33.3)
	≥10	24 (66.7)
Polysubstance dependence	Yes	8 (22.2)
	No	28 (77.8)

*N, number*.

The majority of participants had a very high level of severity of alcohol dependence (66.7%). The cognitive functions are described in Table 2. Mean scores and frequency distribution (percentage) for the global score and for each subtest of ENB-2 Battery were calculated. The 11.1% of AUD participants showed an impaired global score and the 5.7% with scoring at limits of norm. In the sample, only 7 participants scored without impairment in each subtest, six had scores al limits of norm on one or more subtests. Table 2 shows the six cognitive domains assessed by ENB-2. The worst results were in the visuo-spatial abilities and in the executive functions. In particular, we found Copy drawing with the 34% of impaired scores and Overlapping figure test with the 25%, then the Clock drawing (25%) and the TMT-B (13.9%).

**Table 2:** Cognitive functions in early detoxified AUD subjects (*N* = 36).

**Cognitive domain**	**Test**			
		**Mean ± SD**	**Impaired N (%)**	**Limits of norm N (%)**
Attention	TMT A^a^	38.30 ± 16.08	2 (5.6)	–
Memory	Digit span	6.0 ± 1.01	1 (2.8)	1 (2.8)
	Prose memory—immediate recall	15.17 ± 4.27	1 (2.8)	1 (2.8)
	Prose memory—delayed recall	19.28 ± 3.92	–	1 (2.8)
	Memory with interference-−10 seconds	7.42 ± 1.92	3 (8.3)	3 (8.3)
	Memory with interference-−30 seconds	7.08 ± 2.15	3 (8.3)	1 (2.8)
Language	Token test	4.94 ± 0.16	3 (8.3)	1(2.8)
Executive functions	TMT B^a^	127.89 ± 51.37	5 (13.9)	2 (5.6)
	Cognitive estimations	4.83 ± 0.51	1 (2.8)	5 (14.2)
	Abstract reasoning	5.47 ± 0.88	–	1 (2.8)
	Phonemic fluency	12.02 ± 3.86	3 (8.3)	3 (8.3)
	Clock drawing	7.88 ± 2.66	9 (25.0)	3 (8.3)
Visuo-spatial abilities	Overlapping figure test	30.80 ± 8.8	9 (25.0)	4 (11.11)
	Copy drawing	1.64 ± 0.59	12 (34.0)	–
	Spontaneous drawing	1.86 ± 0.42	4 (11.1)	–
Praxis abilities	Ideative and ideomotor praxis test	5.92 ± 0.28	2 (5.6)	–
Global score		77.67 ± 7.86	4 (11.1)	2 (5.7)

a *Mean refers to total time (seconds) used to complete the test; Higher scores, worse performance SD, standard deviation; N, number; %, frequency*.

In Table 3 are shown the mean score and standard deviation of MI abilities and of Comparison cognitive tasks. The global score at MIT did not show pathological scores. Only three participants had scores lower or equal to the 25th percentile. The 30.55% of participants had scores equal to or below the 25th percentile in cognitive performance. In the subscales of MIT only “Clock” and “Cube” tasks had mean scores lower than the average response values for each item.

**Table 3:** Mental Imagery abilities in early detoxified AUD subjects (*N* = 36).

**MIT Battery**	**Test**	**Mean ± SD**	**Range score**	**N. Score ≤ 25^**°**^**
**Variables**				**percentiles (%)**
**MI T Tests**				
Visualizing letters		11.78 ± 0.48	0–12	
Brooks “F” test		8.94 ± 1.94	0–10	
Clock		0.88 ± 1.30	0–4	
Cube		1.61 ± 2.18	0–8	
Subtraction of parts		6.61 ± 3.42	0–12	
Mental exploration of a map		5.77 ± 0.72	0–6	
Imagined paths		7.66 ± 2.71	0–11	
Mental representation of shapes of objects		19.22 ± 1.41	0–20	
**MIT Total score**		**62.75** **±** **9.45**	**0–83**	**3 (8.4%)**
**MIT Comparison cognitive tasks**		
Forward digit span		5.25 ± 0.77	0–6	7 (19.44)
Backward digit span		4.37 ± 1.17	0–6	4 (11.11)
Memory of objects		6.39 ± 0.77	0–7	1 (2.8)
Memory of position of objects		8.42 ± 3.06	0–12	6 (16.7)
Visual-spatial memory test		15.69 ± 2.08	0–18	0
Mirror reproduction of spatial position		15.30 ± 4.27	0–19	1 (2.8)

*SD, standard deviation; N, number*.

### Correlation Analysis

The results of correlational analyses among imagery tasks and perceptual and memory tests showed (Table 4) significant relations between the imagery skills “Subtraction of parts” (*p* < 0.001) and “Imagined paths” (*p* < 0.05) and the cognitive tasks “Visual –spatial memory test” and “Mirror reproduction of spatial position.” Similarly, we found positive significant correlations between “Imagined Paths” and “Forward Digit Span” (*p* < 0.05) and between the “Clock” and “Cube” tasks with “Backward Digit Span” (*p* < 0.05). Finally, we found a positive relationship between “Visualizing letters” and “Memory of positions of objects” (*p* < 0.05). Conversely, the correlations between MI abilities and ENB-2 subscales (Table 5) were the following: the experimental task of “Imagined paths” showed significant correlation with cognitive domains of attention (TMT-A), memory (with interference), executive functions (TMT-B and Clock drawing) and visuo-spatial abilities (overlapping figure test) (*p* < 0.05). “Subtraction of parts” task was related with attention (TMT-A) (*p* < 0.001) and memory functions (Prose memory and memory with interference) (*p* < 0.05). Finally, “Mental exploration of a map” had significant correlations with prose memory performances (*p* < 0.05). We also found a significant relationship between “Brooks F test” and memory (prose) and executive functions (abstract reasoning) (*p* < 0.05) and then between “Cube” task and “Clock drawing” (*p* < 0.05). “Visualizing letters” was related with delayed recall of memory (*p* < 0.05). All correlations were performed with Pearson's coefficient except for 4 subscales of ENB-2 (Clock drawing, Token test, Copy drawing and Praxis test) that did not show normal distribution.

**Table 4:** Correlations among MIT and Comparison tasks.

	**Cognitive comparison tasks**					
**MIT**	**Forward Digit Span**	**Backward Digit Span**	**Memory of objects**	**Memory ofposition of objects**	**Visual-Spatial MT**	**Mirror reproductionof spatial position**
1. Visualizing letters	−0.15	−0.05	−0.07	0.39^*^	−0.04	0.29
2. Brooks “F” test	0.26	−0.09	0.15	0.10	0.02	0.26
3. Clock	0.28	0.34^*^	0.04	0.19	0.16	0.30
4. Cube	0.30	0.37^*^	−0.01	0.19	0.07	0.26
5. Subtraction of parts	0.11	0.31	0.18	−0.14	0.66^**^	0.51^**^
6.Mental exploration of a map	−0.05	−0.04	0.06	–0.35^*^	0.07	−0.05
7. Imagined paths	0.37^*^	0.16	−0.16	−0.06	0.44^*^	0.35^*^
8. Mental representation of shape of objects	0.01	0.02	−0.21	−0.09	0.34^*^	0.08
9. Total Score	0.26	0.23	0.02	−0.09	0.55^**^	0.56^**^

*Data presented as Pearson correlation coefficient (r). In bold*

* *p value < 0.05*;

** *p value < 0.001*.

**Table 5:** Correlations among MIT subscales and ENB-2 subscales.

**MIT**	**ENB-2**											
	**Tot. S**.	**Digit S**.	**PM-IR**	**PM-DR**	**M-10**	**M30**	**TMA**	**TMB**	**Cogn. E**.	**Abstr. R**.	**Ph. Fl**.	**O.F.T**
1. Visualizing letters	0.21	0.01	0.28	0.38^*^	0.01	0.01	−0.23	0.09	−0.03	0.05	0.31	−0.07
2. Brooks “F” test	0.22	−0.09	0.23	0.35^*^	0.12	0.12	−0.15	−0.43^*^	−0.01	0.45^*^	0.09	−0.08
3. Clock	0.10	−0.09	0.11	0.24	0.03	0.03	−0.12	−0.14	0.14	0.22	−0.04	−0.05
4. Cube	0.31	0.07	0.18	0.23	0.29	0.29	−0.27	−0.25	0.14	0.01	0.02	0.14
5. Subtraction of parts	0.60^**^	−0.06	0.27	0.36^*^	0.33^*^	0.33^*^	–0.51^**^	−0.27	0.01	0.12	0.25	0.17
6.Mental exploration of a map	–0.34^*^	−0.07	–0.33^*^	0.33^*^	−0.22	−0.22	0.11	0.21	−0.18	0.03	−0.07	−0.15
7. Imagined paths	0.58^**^	0.17	0.25	0.25	0.40^*^	0.40^*^	–0.42^*^	–0.48^*^	−0.02	0.05	0.21	0.42^*^
8. Mental representation of shape of objects	0.03	0.08	0.10	0.14	−0.06	−0.05	0.01	−0.09	−0.02	−0.13	0.13	−0.02
9. Total score	0.59^**^	−0.09	0.17	0.32	0.40^*^	0.40^*^	–0.49^*^	–0.47^*^	0.03	0.13	0.31	0.26
Data presented as Pearson correlation coefficient (r). In bold ^*^p value < 0.05; ^**^p value < 0.001.												
*Tot S, Total Score; Digit S., Digit Span; PM-IR, Prose Memory-Immediate Recall; PM-DR, Prose Memory-Delayed Recall; M-10, Memory with interference-10 sec; M-30, Memory with interference-30 sec; TMA, Trail Making Test-A, TMB, Trail Making test-B; Cogn. E, Cognitive Estimation; Abstr. R., Abstract Reasoning; Ph. Fl, Phonemic Fluency, O.F.T., Overlapping Figure Test*.												
**MIT**					**ENB-2**							
					**Token t**.		**Clock Dr**		**Copy Dr**		**Praxis t**.	
1. Visualizing letters					0.26		−0.17		0.14		0.39	
2. Brooks “F” test					−0.27		−0.10		0.14		0.34^*^	
3. Clock					0.09		0.10		−0.13		0.22	
4. Cube					−0.28		0.33^*^		−0.19		0.11	
5. Subtraction of parts					–0.34^*^		0.24		0.16		0.45^*^	
6. Mental exploration of a map					−0.14		0.23		−0.20		−0.12	
7. Imagined paths					−0.03		0.39^*^		0.08		0.08	
8. Mental representation of shape of objects					0.07		0.12		−0.03		0.05	
9. Total Score					−0.20		0.35^*^		0.19		0.45^*^	

*Data presented as Spearman correlation coefficient (rho). In bold*

* *p value < 0.05*;

** *p value < 0.001*.

*Token T, Token Test; Clock Dr, Clock drawing; Copy Dr, Copy Drawing; Praxis t, Ideative and Ideomotor praxis test*.

### Regression Model

Multiple linear regression analysis showed (Table 6) that MIT total score was a significant predictor of cognitive functioning (*p* = 0,009) and that the cognitive comparisons tasks of memory and mirror reproduction of spatial position added the 21% of additional variance in the results. The predictors of cognitive performance are expressed in the following result:

**Table 6:** Multiple linear regression between ENB-2 and MIT with comparison tasks.

**ENB-2 Total score**	**Coef**.	**Std. Err**.	**t**	***P***
1. MIT total score	0.3770028	0.1348875	2.79	**0.009**
2. Digit Span (forward and backward)	0.3980384	0.5893266	0.68	0.505
3 Memory (of object and of position of object)	0.7839374	0.2909635	2.69	**0.011**
4.Visual-spatial memory test	−3424349	0.5558705	−0.62	0.543
5.Mirror reproduction of spatial position	0.6281491	0.2696231	2.33	**0.027**
cons	34.35945	9.053555	3.80	0.001
Number of obs = 36				
*F*_(5, 30)_ = 805				
Prob > F = 0.0001				
R-squared = 0.5729				
Adj R-squared = 0.5107				
Root MSE = 5.5508				

*Coef.= regression coefficient; std. err.= standard error coefficient*.

ENB-22(R = 57) = 0.35 MIT total score + 0.13 Memory total score + 0.8 Mirror Reproduction of spatial position.

## Discussion

The present pilot study aimed to examine MI skills in early-detoxified AUD subjects during their residential rehabilitation and to study its relations with the cognitive functions, affected by alcohol-related neuroanatomical alterations. Previous research has shown that AUD is associated with cognitive impairments and that these neuropsychological deficits may influence disease management (4, 43). MI could be considered as a variable underlying many other cognitive functions and influencing overall mental efficiency (24). It's well known that the ability of imagery can be impaired as consequence of brain damage or degenerative syndromes (22, 44). However, in Alzheimer's disease, MI is used to improve memory performance, since that primary visual area tend to remain intact until later during the disease course and that the superiority of pictures remains quite strong in subjects with mild Alzheimer's disease (22). The MI was used, also, as a rehabilitation intervention approach for promoting relearning, after stroke or in people with Parkinson's disease, to improve motor and cognitive functions (25, 27). Our results showed that MI abilities were intact (no subject with total score impaired) compared to the cognitive functions (four participants with global score impaired and two participants in the limits of norm). The subscales with mean scores lower than the average response values for each subscale were “Clock” and “Cube.” Two tasks involving the cognitive processes of inspection, maintenance and manipulation of visual images. Only three participants showed a MIT total score less than or equal to 25th percentile. The cut-off score of MIT for dementia is a total score below the 10th percentile. The cognitive impairments affected predominantly visuospatial abilities, executive functions and memory. Our results are in line with several studies that have demonstrated impaired performance on visuospatial processing, executive functions and episodic memory in AUD subjects (6, 10, 45). The 80.5% of the total sample showed impaired performance on at least one subscale of the neuropsychological battery. Some studies have revealed that specific skills of visualization and image processing may influence performance in the evaluation of overall cognitive functioning and mental deterioration (24, 46). We found significant correlations between MI and the cognitive performance. Particularly, the abilities related to processes of manipulation of visual image (“Subtraction of parts” and “Imagined Paths”) showed significant correlations with attention, memory and executive functions. The processes of generation, inspection and maintenance of visual images were significantly correlated with the cognitive functions of memory. Indeed, Kosslyn et al. (47) suggested that the ability to generate mental representations, in the absence of actual visual input, required the cognitive procedures of generation, inspection, maintenance and manipulation of visual images, in order to manipulate, transform and rotate objects in visual imagery (48). MI is considered a higher brain function (17), involved in reasoning and problem solving, through the retrieval of information from long-term memory stores and the manipulation of such information in working memory (49, 50). Correlational analysis among imagery tasks and perceptual and memory test without generation or manipulation of mental images, showed that MI performances of manipulation of visual images were significant correlated with memory and spatial perception tasks, while other imagery processes resulted significant correlated only with memory tasks. The final goal of this study was to investigate the effects of MI abilities on overall mental efficiency. Our results confirm the significant association between the ability of imagery (MI Test) and the global cognitive performance (ENB-2) in AUD participants and that this strong relationship is greater than the other cognitive tasks (Memory and Mirror Reproduction of Spatial position). The preponderance of imagination over attentional, perceptual and memory tasks on cognitive performance in AUD subjects is similar to effects of MI on cognitive functioning in elderly (46). In literature, the MI ability resulted lower in participants with amnesic Mild Cognitive Impairment and with Alzheimer's disease and the decline was probably referred to the decline of semantic memory (51). In our sample of study, the participants in early alcohol detoxification showed that the functions of MI were substantially preserved compared to the cognitive ones. The lesser impairment of the Imagery compared to general cognitive functioning in alcoholic participants may represent a resource for the residential rehabilitation program. MI is an emerging rehabilitative approach to enhance the motor and cognitive functions in neurorehabilitation (52). Research has shown that Imagery training leads to substantially improvements in memory performance in older adults (53, 54) and in participants with Alzheimer's disease (22). MI has also been applied for promoting memory strategy on recall performance in people with Parkinson's disease (27) and for relearning in subjects after stroke (25). The sensitivity of MIT to detect the cognitive performance in alcohol-dependent subjects and the better performance in MI skills compared to cognitive ones, would allow us to hypothesize the use of strategies of MI to improve the cognitive functions in subjects with AUD and that it could be a useful tool in rehabilitation program. Indeed, MI has been used to induce changes in the affective symptoms of mood and anxiety disorders (28). MI techniques have also shown a significant impact on the reduction of symptoms of central neuropathic pain, highlighting the promising role of MI as rehabilitative approach in improving neuropathic pain (55, 56). Furthermore, imaginative training demonstrated positive effects in facilitating associative learning in Intellectual Disability and in increasing cognitive abilities in atypical development (24, 57). However, the importance of MI for psychiatry extends far beyond the cognitive impairment. It has a strong link to the emotion, serving to modulate emotional states with impact on motivation and behavior (58). This exploratory study has limitations related to the small sample size and further research are needed to improve the validity of results. In particular, studies with larger sample are needed to interpret results of negative correlations among the MIT task “Mental exploration of a map” and some test of ENB-2 and Comparison tasks. In addition, in order to study the changes over time in the cognitive and Imagery performance after detoxification, it would be useful to conduct more than one assessment of cognitive and imagery functions in early- detoxified subjects according to a longitudinal perspective. Finally, a limitation has to be noted as to the multiple regression model. Indeed, the high number of predictors might have led to an increase in type-I error probability; thereupon, we suggest that future studies should focus on run such a model by addressing as predictor(s) global cognitive measures.

## Conclusions

Our results support the importance of the role of MI on cognitive functioning in AUD subjects during early detoxification. Moreover, the better performance in imagery functions respect to cognitive ones could have applicative relevance for rehabilitation from alcohol addiction: the use of Imagery training to improve cognitive performance and rehabilitation intervention.

## Data Availability Statement

The raw data supporting the conclusions of this article will be made available by the authors, upon reasonable request.

## Ethics Statement

The studies involving human participants were reviewed and approved by CER Liguria 387/2018. The patients/participants provided their written informed consent to participate in this study.

## Author Contributions

MO contributed to conception of the study, performed the statistical analysis, and wrote the first draft of the manuscript. ET, CV, and SC contributed to design of study and organized the database and reference. EF and CP wrote sections of the manuscript and organized the tables. All authors contributed to manuscript revision, read, and approved the submitted version.

## Conflict of Interest

The authors declare that the research was conducted in the absence of any commercial or financial relationships that could be construed as a potential conflict of interest.

## Publisher's Note

All claims expressed in this article are solely those of the authors and do not necessarily represent those of their affiliated organizations, or those of the publisher, the editors and the reviewers. Any product that may be evaluated in this article, or claim that may be made by its manufacturer, is not guaranteed or endorsed by the publisher.
